# Can we obtain prognostic information from healthy tissue uptake and volume in baseline ^18^F-FDG PET/CT imaging in diffuse large B-cell lymphoma?

**DOI:** 10.1007/s00259-025-07503-9

**Published:** 2025-08-18

**Authors:** Nienke R. Gerards, Sanne E. Wiegers, Anne L. Bes, Jakoba J. Eertink, Pieternella J. Lugtenburg, Josée M. Zijlstra, Ronald Boellaard, Gerben J.C. Zwezerijnen

**Affiliations:** 1 Department of Radiology and Nuclear Medicine, Amsterdam UMC location Vrije Universiteit Amsterdam, De Boelelaan 1117, 1081 HV Amsterdam, The Netherlands; 2 Cancer Center Amsterdam, Imaging and Biomarkers, Amsterdam, The Netherlands; 3 Department of Haematology, Amsterdam UMC location Vrije Universiteit Amsterdam, Boelelaan 1117, Amsterdam, The Netherlands; 4 Department of Haematology, Erasmus MC Cancer Institute, University Medical Center Rotterdam, Rotterdam, The Netherlands

**Keywords:** Diffuse large b-cell lymphoma, ^18^F-FDG PET/CT, Healthy tissues, Prediction

## Abstract

**Purpose:**

Diffuse large B-cell lymphoma (DLBCL) patients often experience relapse or progression after treatment, emphasizing the need for better prognostic markers. This study investigated baseline FDG uptake and volume of healthy tissues and tumours, to assess their association with time-to-progression (TTP) in DLBCL.

**Methods:**

This study included 259 newly diagnosed DLBCL patients. Outcome was two-year TTP after treatment initiation. Automatic segmentation of tissues was performed on the low dose CT scans. Tumour lesions were outlined with a semi-automated segmentation method (standardised uptake value ≥ 4) and subtracted from the tissues. Mean standardised uptake value (SUVmean), tissue-to-blood SUVmean ratio, volume, and total lesion glycolysis (TLG) were determined for the spleen, liver, kidneys, lungs, and brain. Only SUVmean and SUVmean ratio were assessed for fat, bone, and skeletal muscle. Intercorrelations among all tissues and correlations with tumour TLG were calculated. Volume and uptake measures were compared between patients with and without progression using the Wilcoxon signed-rank test. Any measure significantly associated with two-year TTP was added to an existing logistic regression model based on baseline tumour characteristics to assess its added prognostic value.

**Results:**

Patients with progression showed a significantly higher spleen-to-blood SUVmean ratio, lower SUVmean and TLG in the brain, lower SUVmean in skeletal muscle, and a larger liver volume. Liver volume and spleen-to-blood SUVmean ratio did not significantly improve the prediction of two-year TTP compared to the original model only based on tumour characteristics.

**Conclusion:**

Healthy tissue PET/CT measures were significantly associated with two-year TTP in DLBCL patients. However, these measures did not improve the prediction of two-year TTP compared to models using only tumour characteristics.

**Trial registration number and date of registration:**

HOVON-84: EudraCT: 2006-005, 174 − 42, retrospectively registered 01-08-2008.

**Supplementary Information:**

The online version contains supplementary material available at 10.1007/s00259-025-07503-9.

## Introduction

Diffuse large B-cell lymphoma (DLBCL) is a subtype of non-Hodgkin lymphoma accounting for approximately one-third of non-Hodgkin lymphoma cases worldwide. Standard first-line treatment involves a combination of chemotherapy along with the monoclonal antibody rituximab. Between 25% and 40% of patients receiving this treatment either fail to achieve complete remission or experience relapse [1]. Identifying these patients is important for guiding treatment decisions. The International Prognostic Index (IPI) is the current prognostic tool for DLBCL, but has lost discriminatory power due to the introduction of rituximab [2]. As a result, new biomarkers are needed for early identification of patients at risk for progression or relapse.

^18^F-fluorodeoxyglucose (^18^F-FDG) positron emission tomography (PET) computed tomography (CT) scans at baseline are often used for early prediction of outcome in DLBCL. Quantitative tumour characteristics, such as metabolic tumour volume (MTV) extracted from PET/CT scans, have been frequently published as promising biomarkers for predicting outcome in DLBCL [3–5]. A recent prediction model combining the total MTV, peak standardised uptake value (SUVpeak), and the maximal distance between the largest lesion and any other lesion (Dmax_bulk_) predicted two-year time-to-progression (TTP) significantly better than just the IPI [6]. While this model yielded an AUC of 0.71, the survival rates in the group identified as high-risk were still high. Several other prognostic models were recently published, demonstrating comparable or superior results [7, 8]. Deep learning approaches have also shown promising improvements [9, 10]. Finally. to further improve the prediction of patient outcome in DLBCL from baseline FDG PET/CT studies, recent studies have focused on additional quantitative measures of healthy tumour-free tissues derived from PET/CT scans as possible predictors of outcome [11–19].

Metabolism and volume of tumour-free tissues can possibly be affected by a systemic inflammatory reaction, changes in body composition, or other mechanisms that are not yet understood. In this study, we hypothesise that these factors reflect the overall disease burden, the patient’s physical condition, and may affect patient outcomes. Previous studies demonstrate that an increase in ^18^F-FDG uptake and volume of the spleen is associated with a poor prognosis in other cancer types than DLBCL [11–13]. On the other hand, a decrease in ^18^F-FDG uptake in the cerebellum has been identified as a predictor of poor outcome in DLBCL and other lymphomas [14–16]. Body composition before treatment, such as the loss of skeletal muscle or the amount of intermuscular fat, is also known to influence the prognosis of DLBCL patients [18, 19]. We hypothesised that healthy tissue uptakes in particular the brain, liver, and spleen could have additional prognostic value on top of tumor characteristics [6, 20, 21]. Uptake in skeletal muscle could be a sign of frailty and was therefore also assumed to be possible predictive of outcome.

The aim of this study was to explore the impact of baseline ^18^F-FDG uptake and volume of healthy tissues, meaning tissue devoid of tumour lesions, on two-year TTP in patients with DLBCL. The tissues included in the analysis were the major organs of the body, namely the spleen, liver, kidneys, lungs and brain, as well as tissues related to body composition: fat tissue, skeletal muscle and bone. We used full structural segmentations of these tissues to measure both volume and uptake across the entire tissue. The objectives were to (1) assess correlations between the tissue types, (2) evaluate the correlations between the healthy tissue metrics and quantitative tumour characteristics, (3) examine their associations with two-year TTP, and (4) to assess the added value of these healthy tissue metrics compared to the prognostic model based on tumour characteristics.

## Materials and methods

### Patient population

We evaluated the data of previously untreated DLBCL patients participating in the multicentre randomised phase 3 HOVON-84 study (EudraCT2006-005174-42, NTR1014). This study was approved by the institutional review boards of the participating centres and written informed consent was given by all participants. The inclusion and exclusion criteria of the HOVON-84 trial are detailed elsewhere [22]. Patients from the two treatment arms were combined into one group, as there was no statistically significant difference in TTP between the two arms. The primary outcome measure was two-year TTP, defined as the time between the baseline scan and progression or death, categorised into progression or no progression.

### PET/CT

The participating sites supplied the scans in DICOM format, which were then anonymised. For quality control (QC), the criteria of the EANM guidelines were followed: mean standardised uptake (SUVmean) of the liver should range between 1.3 and 3.0, and plasma glucose should be lower than 11 mmol/L [23]. The details of the QC are published elsewhere [24].

### Image analysis of PET/CT

We collected the SUVmean, tissue-to-blood SUVmean ratio, volume, and total lesion glycolysis (TLG) of the following organs: spleen, liver, brain, kidneys, and lungs. SUVmean and tissue-to-blood SUVmean ratio were also obtained for skeletal muscle, bone, subcutaneous fat, and visceral fat. Volumetric measurements were not collected for these four whole-body structures, because the PET/CT scans did not cover the entire body. All tissues were delineated on the low-dose CT images with the TotalSegmentator tool [25]. The TotalSegmentator tool is an artificial intelligence method that generated whole organ and tissue segmentations from the low dose CT and is publicly available [25]. These CT based segmentations were projected on the PET images and then reviewed and manually adjusted when needed to assure that these segmentation contours followed closely the area of healthy organ uptake. In our paper we opted to use whole organ segmentation in order to obtain healthy organ volume and TLG as well. In some patients, reliable segmentation of one or more anatomical structures was impossible due to artefacts in the CT image, the absence of healthy organ tissue due to lesions, or if a specific organ (e.g. kidney) was absent. If this was the case, the patient was excluded from the dataset. The healthy tissue uptake values were then extracted from the corrected segmentations that were projected on the PET images.

In addition to the anatomical structures, tumour lesions were also outlined using a fully automated preselection (using SUV ≥ 4.0 and volume ≥ 3mL), followed by manual adjustments if necessary. A detailed workflow for this method of lesion delineation has been published elsewhere [26, 27]. Tumour TLG was determined as a measure of tumour load. Subsequently, the tumour volume delineation was removed from the segmentation of the tissues, to obtain a segmentation of only the tumour-free tissue.

Tissue volume and uptake measures were calculated for each tumour-free tissue segmentation. Metrics for bilateral organs (viz. kidneys and lungs) were combined by summing the volume and TLG and calculating the average SUVmean weighted by volume. To correct for possible variation in blood clearance of ^18^F-FDG, the tissue-to-blood SUVmean ratio was included as an additional measure for each anatomical structure. This ratio was calculated by dividing the SUVmean of the structure by the SUVmean of the mediastinal blood pool, which was determined in a volume of interest (VOI) positioned in the ascending aorta.

### Statistical analysis

All statistical analyses were performed using R Studio software version 1.1.456. The Q-Q plots and histograms were inspected to assess the assumptions of normality and homogeneity of variances of all variables. Normally distributed variables were expressed as mean ± SD and non-normally distributed variables as median [25th—75th interquartile range].

To assess intercorrelations among all healthy tissues, Spearman’s rank correlation coefficient was calculated. Additionally, this coefficient was used to evaluate the relationship between tumour TLG and tissue SUVmean, tumour TLG and tissue SUVmean ratio, and tumour TLG and tissue TLG. The Wilcoxon signed-rank test was used to compare healthy tissue PET/CT measures between patients with and without disease progression. Differences were statistically significant if *p* < 0.05.

When a healthy tissue uptake or volume measure differed significantly between patients with and without progression, this measure was incorporated into an existing logistic regression model based only on tumour features to predict two-year TTP. This tumour model was previously published [24] and consisted of the natural logarithm of the total MTV, the natural logarithm of SUVpeak, and the maximal distance between the largest lesion and any other lesion (Dmax_bulk_). The performance of the tumour model and the extended logistic regression model that included healthy tissue measurements in addition to the tumour characteristics were evaluated using repeated stratified five-fold cross-validation with 50 repeats. The cross-validated area under the receiver operating characteristic curve (CV-AUC) was calculated to assess the models. Differences in model performance were statistically analysed using the DeLong test [28].

## Results

### Patient demographics

A total of 373 patients in the HOVON-84 trial underwent a baseline ^18^F-FDG PET/CT scan, and 259 of these were included in our analysis and their baseline characteristics can be found in Table 1. Prior to the segmentation of healthy tissues, patients were excluded for the following reasons: missing essential DICOM information (*n* = 21), out-of-range QC or plasma glucose values (*n* = 20), incomplete PET/CT scans (*n* = 13), no reliable measures of MBP (*n* = 3), or the absence of FDG avid lesions (*n* = 2). In addition, 14 patients who died within two years without signs of progression and 7 patients lost to follow-up within two years were excluded. During the segmentation step, patients were further excluded if there was a complete absence of healthy tissue in at least one tissue due to massive tumour infiltration or if an organ (e.g. spleen or kidney) was missing (*n* = 18). Other exclusion criteria included severe CT artefacts or low CT quality that prevented accurate segmentation (*n* = 12), and errors in the segmentation tool (*n* = 3). A detailed overview of the inclusion process is provided in Supplementary Material 1.

Analyses of the brain were performed on a subgroup of 145 patients, because the brain was not (completely) inside the field of view in the remaining scans.

**Table 1:** Patient demographics

Characteristic	No progression	Progression	*p*-value
Number of patients	214 (83%)	45 (17%)	
Sex (male)	106 (50%)	25 (56%)	0.57
Length (cm)	171.3 (± 9.61)	172.0 (± 9.46)	0.67
Weight	77 [67–85]	72 [61–85]	0.17
Age at diagnosis			
Median	65 [55–72]	64 [61–72]	0.83
> 60	142 (66%)	34 (76%)	0.30
WHO performance status score			
0	136 (63.6%)	17 (37.7%)	< 0.01**
1	56 (26.2%)	19 (42.2%)	
2	21 (9.8%)	8 (17.7%)	
Unknown	1 (0.5%)	1 (2.2%)	
IPI			
Low	41 (19.2%)	3 (6.7%)	< 0.01**
Low-intermediate	62 (29.0%)	4 (8.9%)	
High-intermediate	64 (29.9%)	22 (48.9%)	
High	47 (22.0%)	16 (35.6%)	

Dichotomous variables are described as n (%), continuous variables with a normal distribution as mean (±standard deviation), and continuous variables with a non-normal distribution as median [interquartile range]. Significant differences are indicated by ** (*p*<0.01). *Abbreviations:* *WHO* World Health Organization, *IPI* International Prognostic Index

Dichotomous variables are described as n (%), continuous variables with a normal distribution as mean (± standard deviation), and continuous variables with a non-normal distribution as median [interquartile range]. Significant differences are indicated by ** (*p* < 0.01). *Abbreviations*: WHO, World Health Organization; IPI, International Prognostic Index.

### Correlations between healthy tissues

**Fig. 1:**
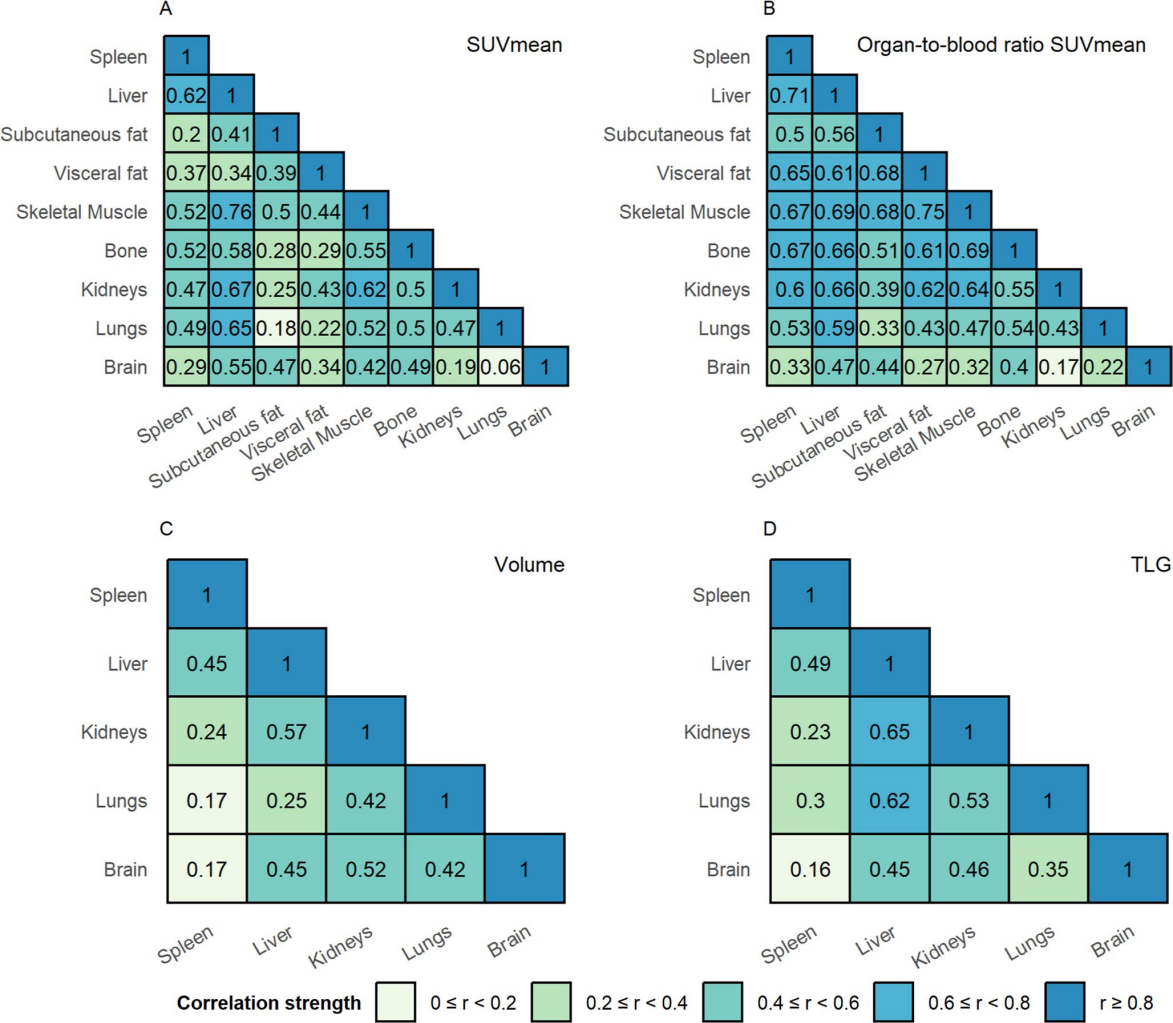
Intercorrelations between the SUVmean, tissue-to-blood SUVmean ratio, TLG, and volume of different healthy tissues. Volume and TLG are only determined in organs that are completely in the FOV. For all measures, the Spearman rank correlation coefficient is determined and for visualization divided into five categories: (1) 0 < *r* < 0.20, (2) 0.20 ≤ *r* < 0.40, (3) 0.40 ≤ *r* < 0.60, (4) 0.60 ≤ *r* < 0.80, and (5) *r* ≥ 0.80. *Abbreviations*: *SUVmean *Mean Standardised Uptake Value, *TLG *Total Lesion Glycolysis, *FOV *field of view

Positive correlations were observed between all tissues for SUVmean, tissue-to-blood SUVmean ratio, volume, and TLG (Fig. 1). However, the strength of these correlation, as measured by Spearman’s rank correlation coefficient, varied. Correlations between the SUVmean of the tissues ranged from 0.06 to 0.76, with the strongest correlations between the liver and skeletal muscle (*r* = 0.76, *p* < 0.001), liver and kidneys (*r* = 0.67, *p* < 0.001), and liver and lungs (*r* = 0.65, *p* < 0.001) (Fig. 1A). When normalised to the mediastinal blood pool SUVmean, correlations between different tissues generally became stronger, except for those with the brain, which remained similar or slightly decreased (Fig. 1C). Correlations between tissue volumes ranged from 0.17 to 0.57 (Fig. 1C). Finally, TLG, which has a SUVmean component besides a volume component, showed a range in correlation strength between 0.15 and 0.65, with the strongest correlations between the liver and kidneys (*r* = 0.65, *p* < 0.001) and liver and lungs (*r* = 0.62, *p* < 0.001) (Fig. 1D).

### Correlations with tumour TLG

**Fig. 2:**
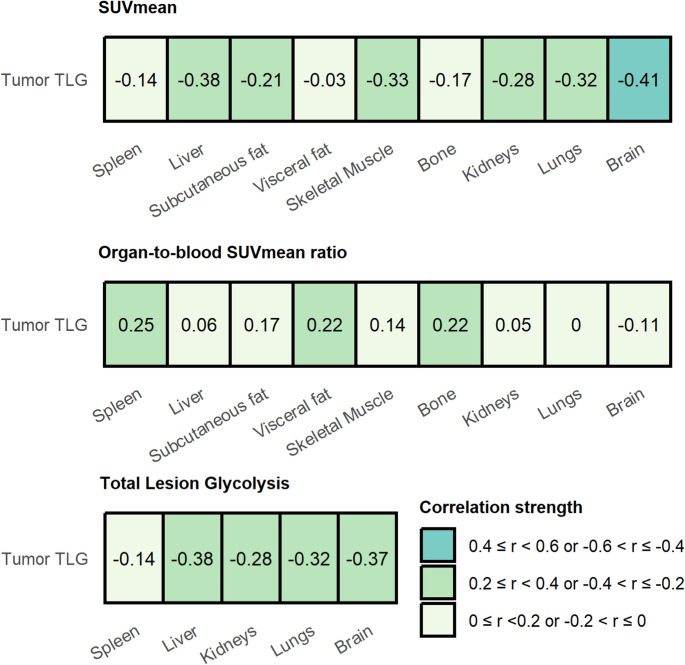
Correlations between tumour TLG and tissue SUVmean, tissue-to-blood SUVmean ratio, and TLG. The Spearman rank correlation coefficient is determined and for visualization divided into three categories: (1) 0.40 ≤ *r* < 0.60 or −0.60 ≤ *r*<−0.40, (2) 0.20 ≤ *r* < 0.40 or −0.40 ≤ *r*<−0.20, and (3) 0 < *r* < 0.20 or −0.20 ≤ *r* < 0. *Abbreviations*: *TLG *Total Lesion Glycolysis, *SUVmean *Mean Standardised Uptake Value

Figure 2 shows the correlations between tumour TLG and the SUVmean, SUVmean ratio, and TLG of the healthy tissues. Tumour TLG was negatively correlated with the SUVmean and TLG of all tissues, with the strongest correlation between tumour TLG and brain SUVmean (*r*=−0.41, *p* < 0.001). The tissue-to-blood SUVmean ratios demonstrated a weak positive correlation with tumour TLG, except for the brain-to-blood SUVmean ratio (*r*=−0.11, *p* = 0.19.

### Associations between PET/CT measures and time-to-progression

**Fig. 3:**
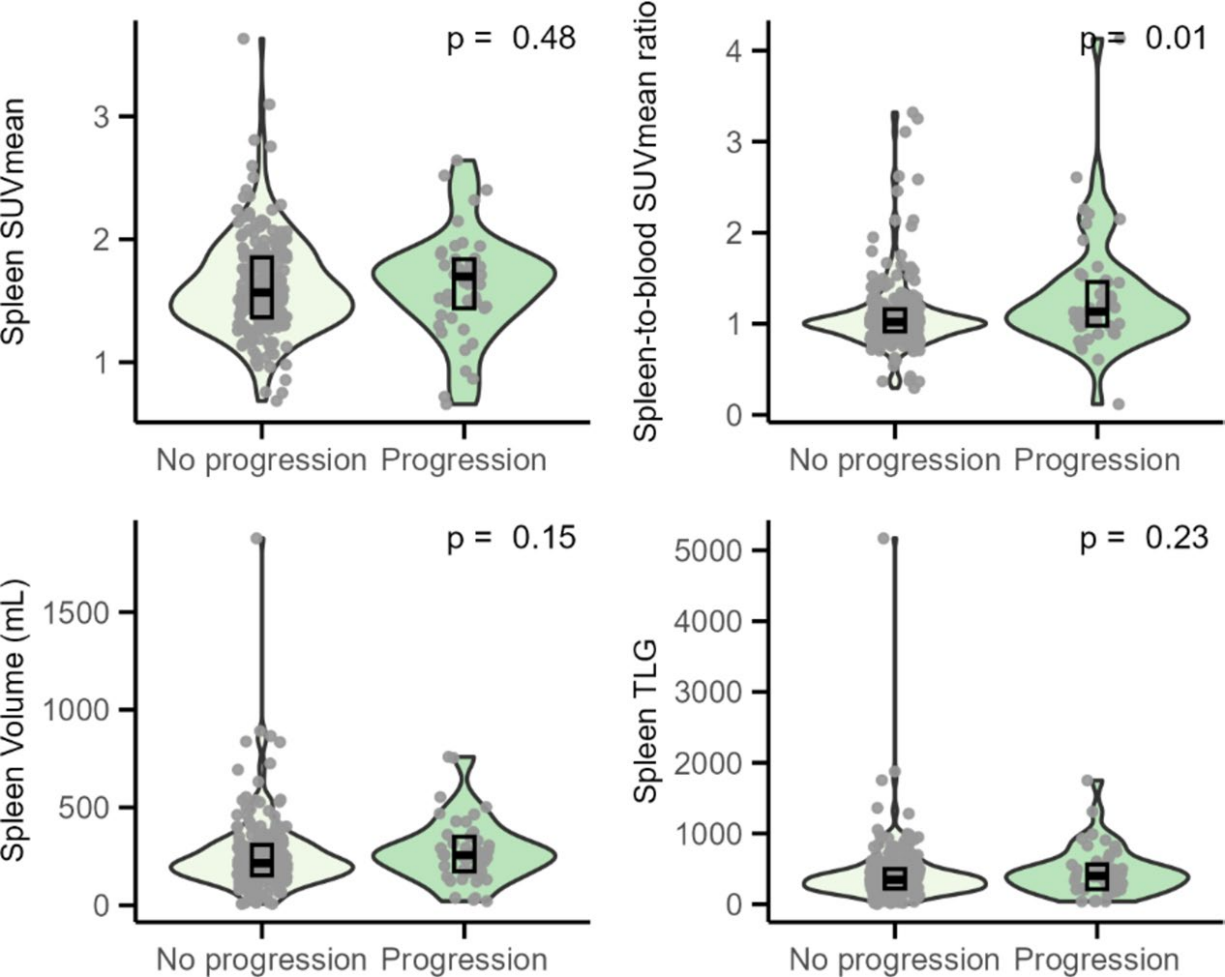
Associations between splenic ^18^F-FDG uptake and volume measures and two-year time-to-progression in DLBCL patients. Differences were statistically significant if p<0.05. *Abbreviations: SUVmean* Mean Standardised Uptake Value, *mL *millilitre, *TLG *Total Lesion Glycolysis

**Fig. 4:**
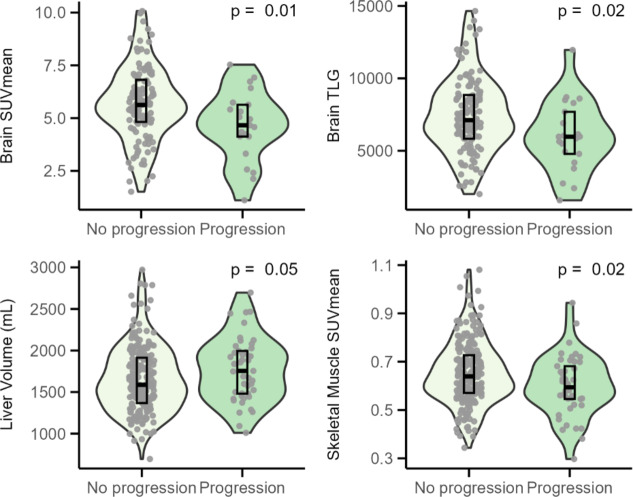
Statistically significant differences (*p* < 0.05) in ^18^F-FDG uptake and volume measures of healthy tissues between DLBCL patients with and without progressing disease within two years. *Abbreviations*: *SUVmean *Mean Standardised Uptake Value, *TLG *Total Lesion Glycolysis, *mL *milliliter

Table 2 reports the median SUVmean, tissue-to-blood SUVmean ratio, volume, and TLG of healthy tissues in patients with and without progressing DLBCL. In the spleen, only an increased spleen-to-blood SUVmean ratio was significantly associated with two-year TTP (Z = 3562, *p* < 0.01), while the other measures showed no significant associations (Fig. 3). Figure 4 shows the statistically significant differences that were observed in the other tissues. The SUVmean and TLG in the brain were significantly lower in patients with progression (Z = 1723, *p* = 0.01; Z = 1714, *p* = 0.02) (Fig. 4AB) and a decreased SUVmean in skeletal muscle was also associated with progression (Z = 5885, *p* = 0.02) (Fig. 4C). Volume of the liver was significantly larger in patients with progression (Z = 3918, *p* = 0.05) (Fig. 4). In all significant associations, however, the group differences remained small, and the within-group variability was high.

**Table 2:** Associations between healthy tissue ^18^F-FDG uptake and volume and two-year time-to-progression

	SUVmean			Tissue-to-blood SUVmean ratio		
Anatomical structure	*TTP > 2y*	*TTP ≤ 2y*	*p-value*	*TTP > 2y*	*TTP ≤ 2y*	*p-value*
Spleen	1.57 [1.37–1.85]	1.70 [1.44–1.84]	0.48	**1.02** **[0.92–1.16]**	**1.13** **[0.98–1.46]**	< 0.01**
Liver	1.85 [1.57–2.12]	1.84 [1.50–2.01]	0.25	1.23 [1.11–1.34]	1.25 [1.15–1.47]	0.10
Brain	**5.62** **[4.82–6.81]**	**4.66** **[4.13–5.63]**	0.01*	3.50 [2.90–4.25]	3.03 [2.53–3.93]	0.32
Kidneys	2.19 [1.95–2.57]	2.22 [1.82–2.60]	0.94	1.45 [1.31–1.69]	1.53 [1.33–1.93]	0.20
Lungs	0.57 [0.47–0.68]	0.54 [0.47–0.65]	0.36	0.37 [0.32–0.44]	0.39 [0.33–0.47]	0.23
Subcutaneous fat	0.38 [0.32–0.44]	0.37 [0.29–0.43]	0.46	0.25 [0.21–0.29]	0.27 [0.22–0.35]	0.13
Visceral fat	0.81 [0.70–0.95]	0.82 [0.70–0.99]	0.82	0.53 [0.45–0.64]	0.58 [0.45–0.82]	0.08
Skeletal muscle	**0.64** **[0.57–0.73]**	**0.59** **[0.55–0.68]**	0.02*	0.42 [0.38–0.48]	0.42 [0.36–0.52]	0.81
Bone	0.95 [0.83–1.15]	0.93 [0.81–1.12]	0.72	0.64 [0.55–0.75]	0.67 [0.54–0.83]	0.28
	Volume			Total Lesion Glycolysis		
Spleen	215.1 [153.1-308.9]	256.2 [173.0-350.6]	0.15	347.5 [222.5-498.3]	400.8 [214.0-563.2]	0.23
Liver	**1587.5** **[1368.2-913.3]**	**1745.5** **[1480.3-995.1]**	0.05*	2989.3 [2340.3-567.3]	3091.8 [2337.6-770.3]	0.61
Brain	1305.3 [1185.6-1379.3]	1291.9 [1216.4-1368.1]	0.95	**7113.5** **[5832.0-850.2]**	**5969.3** **[4784.8-687.4]**	0.02*
Kidneys	321.2 [246.9-363.6]	298.8 [250.1-337.5]	0.10	689.7 [547.7-864.5]	616.2 [469.8-805.5]	0.11
Lungs	3069.9 [2504.9-776.3]	3225.4 [2490.4-3723.8]	0.71	1715.3 [1404.1–2121.0]	1746.8 [1335.9-2015.8]	0.50

Variables are described as median [interquartile range]. Significant differences are indicated by * (*p*<0.05) or ** (*p*<0.01). *Abbreviations:* *SUVmean* Mean Standardised Uptake Value, *TTP* Time-to-progression, *2y* two-year

### Added value of healthy tissue measures

**Fig. 5:**
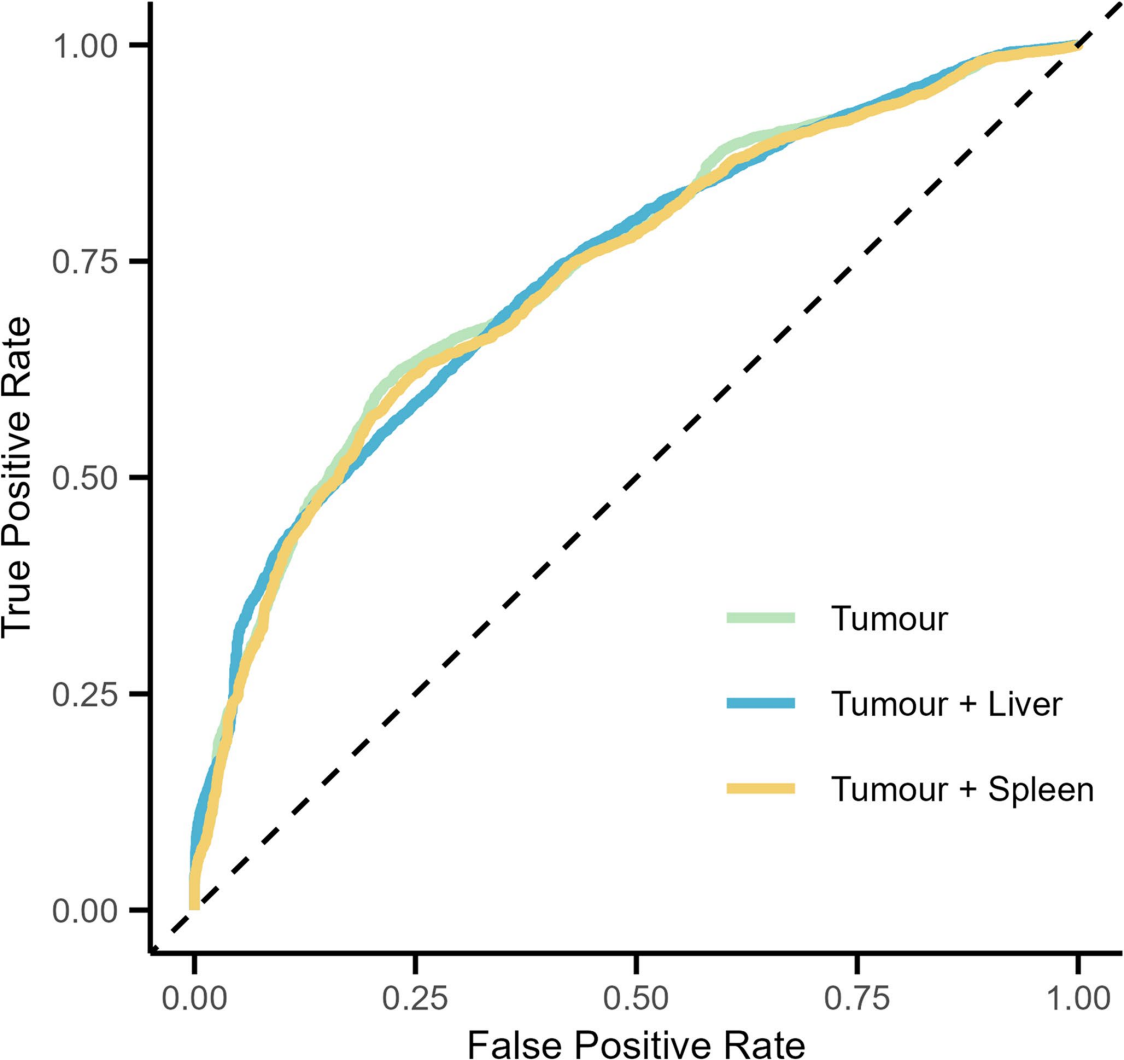
Receiver operating characteristics curves for two-year time-to-progression for the tumour model (metabolic tumour volume [MTV], peak standardised uptake value [SUVpeak], and maximal distance between the largest lesion and any other lesion [DMax_bulk_]), the tumour model along with liver volume, and the tumour volume along with spleen-to-blood SUVmean ratio

Liver volume and spleen-to-blood SUVmean ratio were added individually to the tumour model based on the total MTV, SUVpeak, and Dmax_bulk_. The original tumour model demonstrated an AUC of 0.75. The AUC of the liver model was 0.74, and the spleen model also showed an AUC of 0.74. No statistically significant differences were found in the ROC of the tumour model compared to those of the liver and spleen models (Fig. 5).

## Discussion

This exploratory study demonstrated that patients with progressing DLBCL have higher normalised splenic uptake, lower brain and skeletal muscle uptake, and larger liver volume at baseline. However, these healthy tissue PET/CT measures did not improve prediction of patients at risk of progression when added to an existing model that used tumour uptake characteristics only.

The spleen is affected in 30%−40% of Non-Hodgkin lymphoma patients and shows up on PET/CT scans as either focal lesions or diffuse uptake [29]. Focal lesions were removed in our analysis, so the increased splenic uptake observed in patients with progression is likely attributed to a higher diffuse metabolism in the spleen, which is an indication of lymphomatous involvement according to the Lugano classification [30, 31]. Similar to our results, previous research in other cancer types has shown that diffuse splenic uptake, measured by the spleen-to-liver ratio, is associated with poor prognosis [12, 13]. In Hodgkin lymphoma patients, the splenic SUVmean without normalisation was not associated with TTP [17], which is consistent with our findings. This suggests that normalisation of the splenic uptake with a reference value, such as the liver or mediastinal blood pool is necessary to correct for technical inconsistencies and take into account the distribution of FDG over the body.

In line with our results, previous studies of DLBCL and other lymphoma types also reported an association between lower uptake in the brain and disease progression. Godard et al. [14] found in a cohort of 46 DLBCL patients that the ratio of cerebellar SUVmean to hepatic SUVmean was significantly lower in patients with progression within two years compared to those without. Similarly, Morland et al. [15, 16] demonstrated that a model incorporating cerebellar SUVmax and hepatic SUVmean was as effective as total MTV in predicting survival in Hodgkin lymphoma in 163 DLBCL patients. We have now demonstrated that the associations between lower uptake in the brain and disease progression remain consistent in a larger cohort.

It is speculated that reduced uptake in healthy tissues may be linked to poor prognosis due to ‘metabolic theft’ by the tumour, which deprives other tissues of available FDG. Bulky tumour disease - characterised by a tumour diameter exceeding 10 cm - is more prevalent in patients with poorer outcomes [32]. This likely explains the lower uptake observed in the tissues of patients with progressing disease. Further supporting the metabolic theft hypothesis, we observed a negative correlation between tumour TLG and brain uptake, which is consistent with the findings of Hanaoka et al. [33]. The SUVmean of other tissues was also negatively correlated with tumour TLG, with the strongest correlations observed with the liver, skeletal muscle, and lungs. When normalised for tracer availability in the blood, the negative correlations of all tissues with tumour TLG decreased or even became positive. Together, this shows that metabolic competition between healthy tissues and tumours is likely an important factor in the observed group differences in the brain and skeletal muscle. In the spleen, metabolic theft may be counterbalanced by intrinsic changes in metabolism, potentially cancelling each other out. This could explain why a difference between patients with and without progression was only observed in the normalised splenic SUVmean, but not in the non-normalised splenic SUVmean.

Our results highlight the importance of normalising tissue uptake to a reliable reference for background uptake. Typically, the SUVmean of the liver or mediastinal blood pool is used, as these values have been shown to remain stable over time in a cancer-free population [34]. In this study, we opted for the mediastinal blood pool, given the potential effect of cancer on liver metabolism. Normalisation to the blood pool not only minimises technical variability in the data but also helps rule out metabolic competition in cases of high tumour burden. This approach reduces the risk of false positive associations between FDG uptake in healthy tissues and disease progression.

Various other factors can also influence intrinsic metabolic changes within the body. One such factor is age, which is associated with a reduced cerebral metabolism. This is particularly relevant in our cohort, because the patients with progressing disease are older on average [35]. Specific medications, such as corticosteroids, benzodiazepines, and neuroleptic drugs are also known to reduce the global glucose metabolism of the brain significantly [36]. In skeletal muscle, ^18^F-FDG accumulation is seen in diabetes patients or after exercise [37]. Unfortunately, our dataset was not sufficient to include medication or other comorbidities as confounding factors in the analysis.

The previously published logistic regression model based on tumour characteristics including the total MTV, SUVpeak, and Dmax_bulk_, showed comparable performance in our selected patient cohort as in the original study [6]. Its performance was also comparable to other prognostic models based on tumour characteristics only [20, 21]. Extending the logistic regression model with splenic uptake and liver volume did not improve the prediction of two-year TTP, likely due to relatively week associations with outcome as well as the large within-group variability. Our prognostic model also showed comparable, yet slightly lower, performance with a deep learning based model by Jiang et al. [10], which showed potential improvements in prognoses when using deep learning methods. Brain and skeletal muscle uptake measures were not included in the tumour model due to their correlations with tumour uptake, which could result in multicollinearity in the prediction model. Our findings suggest that healthy tissue metabolism is influenced by multiple factors, and may not provide consistent prognostic value in DLBCL. As a result, future efforts may be better directed toward integrating other data types, such as such as histopathological features, genetic information, or circulating tumor DNA into prognostic models.

This study also investigated the intercorrelations of PET/CT measures between tissues. Assessing organ systems rather than individual structures may provide a more complete view of complex pathologies like DLBCL, as network interactions might be disrupted [38]. We observed strong correlations in tissue-to-blood SUVmean ratios across most healthy tissues. However, a limitation was that, since static PET acquisition was used, metabolic relationships could only be evaluated at group level and not for individual patients. To solve this issue, Sun et al. [39] proposed a framework comparing disease and reference networks to reflect the variation between normal and diseased systems. Applying this network model, or using dynamic instead of static scans [40], could provide more insights into organ connectivity in our population in the future.

Limitations of the study were its retrospective design and the number of statistical tests, which increased the probability of obtaining at least one statistically significant test by chance. No adjustment for multiple comparisons was made, as the study was exploratory. In addition, the accuracy of the healthy tissue measures may sometimes be hampered by signal spill over from surrounding tissues, like visceral fat bordering high-uptake organs like the liver, kidneys, spleen, and bladder. However, we tried to minimise this effect to the best of our ability through manual adjustments to the tissue segmentations. Alternatively, small (< 3 cm diameter spherical) fixed-size VOIs can be used to derive healthy tissue SUV [17]. This avoids signal spillover but would not allow to assess whole organ volume or TLG. Additional analysis using fixed size VOIs revealed that our whole organ VOIs provided liver and splenic SUVmean within 5% of those seen with fixed sized VOIs. Yet, fixed sized VOI are a more widely available and easy to use approach avoiding the need for an AI based method. Because of the exploratory nature of this study, our results require a more extensive validation on other datasets. Within the PETRA consortium, prognostic models including spleen ^18^F-FDG uptake are currently under investigation using a different and larger cohort. To our knowledge, this study is the largest investigating the associations of uptake and volume determined in full structural segmentations of several major healthy organs and tissues and TTP in DLBCL.

## Conclusion

Patients with progressing DLBCL show lower brain and skeletal muscle ^18^F-FDG uptake, higher splenic ^18^F-FDG uptake, and larger liver volume. However, these healthy tissue PET/CT measures did not improve prediction of patients at risk of progression compared to models that include tumour uptake characteristics only.

## Supplementary Information

Below is the link to the electronic supplementary material.

ESM1 (27.0 KB)

## Data Availability

The datasets generated during and/or analysed during the current study are available from the corresponding author on reasonable request and considering the approval given by the ethics review boards and patients.
