# Supplementary material for: Can we obtain prognostic information from healthy tissue uptake and volume in baseline 18F-FDG PET/CT imaging in diffuse large B-cell lymphoma?
Source: Eur J Nucl Med Mol Imaging. 2025 Aug 18;53(2):1053–63. doi: 10.1007/s00259-025-07503-9 (PMC12830424; doi:10.1007/s00259-025-07503-9)
Supplement: Supplementary file 1 — ESM1 (27.0 KB) [file 259_2025_7503_MOESM1_ESM.docx]

**Title:** Can we obtain prognostic information from healthy tissue uptake and volume in baseline ^18^F-FDG PET/CT imaging in Diffuse Large B-cell Lymphoma?

**Journal:** European Journal of Nuclear Medicine and Molecular Imaging

**Authors and Affiliations:** Nienke R. Gerards^1,2^*, Sanne E. Wiegers^2,3^, Anne L. Bes^2,3^, Jakoba J. Eertink^2,3^, Pieternella J. Lugtenburg^4^, Josée M. Zijlstra^2,3^, Ronald Boellaard^1,2^, Gerben J.C. Zwezerijnen^1,2^

*^1^Amsterdam UMC location Vrije Universiteit Amsterdam, Department of Radiology and Nuclear Medicine, De Boelelaan 1117, Amsterdam, The Netherlands*

*^2^Cancer Center Amsterdam, Imaging and Biomarkers, Amsterdam, The Netherlands*

*^3^Amsterdam UMC location Vrije Universiteit Amsterdam, Department of Haematology, Boelelaan 1117, Amsterdam, The Netherlands*

*^4^Department of Haematology, Erasmus MC Cancer Institute, University Medical Center Rotterdam, Rotterdam, The Netherlands*

*Corresponding author and first author: Nienke R. Gerards, De Boelelaan 1117 1081 HV Amsterdam, The Netherlands, E-mail address: [n.r.gerards@amsterdamumc.nl](mailto:n.r.gerards@amsterdamumc.nl).

**Supplementary material 1**

**Fig. 1** This figure shows an overview of the exclusion criteria. In the first round of exclusion, patients were excluded if baseline ^18^F-FDG PET/CT scans were incomplete, failed QC, or lacked identifiable tumour lesions. Additionally, patients were excluded if it was impossible to make a reliable region of interest in the mediastinal blood pool due to bad CT quality in the area of the ascending aorta. Patients who died within two years without progression, and who were lost to follow-up were also excluded. In the second exclusion round, patients without a complete set of tissue segmentations were excluded. The primary reason for an incomplete dataset was massive tumour infiltration preventing segmentation of healthy tissue in at least one of the structures. Severe CT artefacts or a poor CT quality also hindered tissue segmentation. One patient had no identifiable spleen and one patients was missing one kidney. Lastly, an error in the quantification tools led to the exclusion of three patients. Out of the 259 patients included in this analysis, 145 had the brain completely inside the field of view of the scanner. These patients were used for the analysis of brain PET/CT measures. *Abbreviations:* QC, Quality Check; WB/TB, Whole-body/Total-body.

Patients in Hovon-84 trial

n = 373

Patients with complete baseline ^18^F-FDG PET/CT and clinical outcome data

n = 292

Patients with complete organ segmentations

n = 259

Brain subset with brain completely in field of view

n = 145

- Missing Dicom information n = 21
- QC outside of range n = 20
- Incomplete WB/TB scan n = 13
- Mediastinal blood pool not reliable n = 4
- No FDG avid lesions n = 2
- Death without progression n = 14
- Lost to follow-up n = 7
- Massive tumour infiltration n = 16
- Severe CT artefacts n = 10
- Bad CT quality n = 2
- Missing organ n = 2
- Error in tools n = 3
